# Parental Knowledge and Attitudes Toward Emergency Management of Dental Trauma in Children: A Cross-Sectional Croatian Study

**DOI:** 10.3390/pediatric18010011

**Published:** 2026-01-15

**Authors:** Klaudia Aleric, Lidia Gavic, Mirna Draganja, Kristina Gorseta, Vesna Ambarkova, Antonija Tadin

**Affiliations:** 1 School of Dental Medicine, University of Zagreb, 10000 Zagreb, Croatia; kaleric@sfzg.hr; 2 Department of Endodontics and Restorative Dental Medicine, Study of Dental Medicine, School of Medicine, University of Split, 21000 Split, Croatia; 3 Private Dental Practice, 4811 Breda, The Netherlands; mirna_draganja@yahoo.com; 4 Department of Pediatric and Preventive Dentistry, School of Dental Medicine, University of Zagreb, 10000 Zagreb, Croatia; kgorseta@sfzg.hr; 5 Department of Pediatric and Preventive Dentistry, Faculty of Dental Medicine, Saints Cyril and Methodius University, 1000 Skopje, North Macedonia; vambarkova@stomfak.ukim.edu.mk; 6 Department of Dental Medicine, University Hospital of Split, 21000 Split, Croatia

**Keywords:** tooth injuries, parents, knowledge, emergency treatment, pediatric dentistry, tooth avulsion, mouth protectors, cross-sectional studies

## Abstract

*Aim*: Traumatic dental injuries (TDI) in children are a common but often underestimated emergency. Parental knowledge and timely response are crucial for successful treatment. This study aimed to evaluate parental knowledge, experiences, and awareness regarding dental trauma management and the use of protective mouthguards. *Methods:* A cross-sectional study was conducted using a self-administered questionnaire among 333 parents in dental clinics in Split and Zagreb, Croatia. The questionnaire assessed sociodemographic data, parental knowledge of TDIs, and prior experience with dental trauma. Statistical analysis included chi-square test (*p* < 0.05). *Results:* The overall level of parental knowledge regarding traumatic dental injuries was generally low (7.6 out of 15 points). Almost all parents correctly identified the age when children have primary or permanent teeth. However, less than half knew that an avulsed primary tooth should not be replanted, while about three-quarters recognized that professional help should be sought within 30 min after trauma. Overall, 43.5% of parents reported that their child had experienced dental trauma, most often affecting primary teeth (60.7%), particularly the maxillary central incisor (76.6%). Mothers demonstrated significantly higher knowledge than fathers (*p* = 0.025), and prior experience or information about dental trauma significantly improved awareness (*p* < 0.001). Although 54.3% of respondents were unaware of the purpose of dental shields, 82.3% considered them necessary during contact sports, yet only 12.9% reported that their child actually uses them. *Conclusions:* Within the limitations of this clinic-based study, the findings indicate gaps in parental knowledge regarding the appropriate management of dental trauma. Strengthening parents’ understanding of emergency response and preventive measures may support timelier and appropriate care and contribute to improved outcomes for children experiencing traumatic dental injuries.

## 1. Introduction

The definition of dental trauma is extremely broad, as it refers to any lesion affecting the dentition (the hard or soft tissues of the tooth, the pulp, or the periodontal structures). Any thermal, mechanical or chemical lesion that damages a tooth is referred to as a traumatic dental injury (TDI) [1].

Traumatic dental injuries (TDIs) in children are increasingly becoming a significant dental public health problem worldwide [2]. The prevalence of dental injuries varies widely, ranging from 6% to 59%. Although there are variations, studies generally show that approximately one-third of preschool children (primary teeth) and one-quarter of adolescents and adults (permanent teeth) have experienced dental trauma at least once in their lifetime [3]. Studies in Croatia have shown that the most affected permanent tooth in dental trauma is the maxillary central incisor (80.9%) [4,5].

If a TDI occurs, the prognosis is significantly improved when the traumatized tooth is properly managed immediately at the accident site, right after the dental trauma [6]. Early management is often based on the comprehension of parents who happen to be near at the moment of unfortunate incident. Previous studies indicate people have little understanding regarding first aid of traumatized teeth [7,8,9]. It is the responsibility of the dental profession to provide public education about the risks of sports-related TDIs and the preventive measures that can be taken [10,11,12]. The most effective prevention strategy involves not only education about primary prevention but also ensuring proper secondary prevention, which requires prompt and appropriate management in the event of an injury [10,11,13]. Previous research on parents’ knowledge of dental trauma has revealed a limited knowledge about TDIs [14,15]. Should the parents’ knowledge of appropriate care and action at the time of DT be lacking, the likelihood of a poor outcome increases. The likelihood of a poor TDI outcome increases with the lack of parents’ knowledge about the appropriate care and course of actions when the dental trauma happens. Hence, it is extremely important that parents know how to act in case of dental injuries [16,17,18]. Furthermore, there is a need to intensify oral health education targeted at parents to inform them about the consequences of traumatic dental injuries and to teach them how to react properly in the event of TDIs.

There is no research on parents’ knowledge of dental trauma in Croatia, but there are studies that have investigated the knowledge among teachers, pediatricians, and athletes [19,20,21,22]. The aim of this study was to assess the awareness and knowledge of parents attending dental clinics in Split and Zagreb regarding traumatic dental injuries and their emergency management in children. The secondary aim was to identify factors associated with parental knowledge, including previous education, prior experience with traumatic dental injuries, and selected sociodemographic characteristics.

## 2. Population and Methods

### 2.1. Study Design and Ethical Considerations

This descriptive, cross-sectional study was conducted using a self-administered paper-based questionnaire between February and April 2025. The study involved parents whose children were patients at two institutions: the Dental Polyclinic Split and the Department of Pediatric Dental Medicine at the University of Zagreb School of Dental Medicine, Croatia. The study followed the STROBE guidelines for cross-sectional research [23]. Participation was voluntary and anonymous. Ethical approval was obtained from the Ethics Committee of the University of Split School of Medicine on 23 January 2025 (Approval No.: 2181-198-03-04-25-0005; Class No.: 003-08/20-03/0005).

Participation was voluntary and anonymous. Prior to participation, all parents provided written informed consent. The consent form informed participants about the purpose of the study, the identity of the investigators, and the anonymous handling of data. No incentives were offered for participation.

### 2.2. Participants and Eligibility Criteria

A convenience sample of parents or legal guardians of pediatric patients aged up to 18 years was recruited at two large dental polyclinics in Split and Zagreb. Participants were approached in the clinic waiting rooms by members of the research team and invited to participate in the study while awaiting their child’s dental appointment. Data collection was conducted on site using an anonymous self-administered questionnaire. As a clinic-based sample, the study population reflects parents attending dental services rather than the general parent population.

Inclusion criteria were parents/legal guardians of children under 18 receiving treatment at the participating institutions; ability to read and understand Croatian; residency in Croatia; willingness to provide informed consent. While exclusion criteria were: incomplete or partially filled questionnaires; parents who were dental professionals (to avoid bias from professional knowledge); parents of children with diagnosed intellectual or developmental disabilities unrelated to dental trauma; inability to recall or provide relevant information due to language or cognitive difficulties; and parents who were not the primary caregivers. All questionnaires that met the inclusion criteria were fully completed and included in the final analysis (*n* = 333).

### 2.3. Questionnaire Instrument

The questionnaire was constructed based on a literature review of dental trauma knowledge and parental preparedness and was reviewed by two domain experts in pediatric and restorative dentistry [7,8,9,14]. The questionnaire was developed by two pediatric dentistry specialists and two general dental practitioners, who designed and reviewed its content and structure. It consisted of 40 questions divided into five sections, covering knowledge and experience about dental trauma and management of dental trauma. A pilot study was conducted with 20 parents to evaluate clarity, usability, and feasibility; participants confirmed the readability and suggested minor revisions. Their responses were not included in the final study sample. Following the pilot, the questionnaire was approved by specialists in endodontics and pediatric dentistry. The questionnaire consisted of two main domains: a parental sociodemographic domain and a knowledge assessment domain. The parental sociodemographic data domain (9 items) included questions on age, gender, residence, number of children, education level, employment, marital status, income, and healthcare background. As these variables are descriptive and do not represent a latent construct, internal consistency analysis was not applicable to this domain.

The knowledge assessment domain (12 items) consisted of multiple-choice questions evaluating parental knowledge of emergency management of dental trauma (e.g., avulsion, crown fracture). Nine items were scored as 1 point each, while three key items were weighted at 2 points, resulting in a maximum possible score of 15 points. The internal consistency of the knowledge assessment items was evaluated using Cronbach’s alpha (α = 0.743), indicating acceptable internal consistency. Based on Bloom’s cut-off points, knowledge was classified as “good knowledge” for participants scoring 80% or more (12 to 15 points), while individuals scoring between 60 and 79% (9 to 11.85 points) were designated as having “moderate knowledge”. Conversely, respondents scoring below 60% (below 9 points) were identified as having “poor knowledge” within the context of this study [24]. The next section of the questionnaire included four items assessing the child’s experience with dental trauma, witnessing of dental injuries, and the parents’ awareness of and self-assessed knowledge regarding dental trauma. The experience with trauma domain (11 items) targeted parents whose children had sustained dental trauma and gathered information on the type of trauma, type of tooth involved (primary or permanent), circumstances of the injury, and tooth vitality. The mouthguard awareness and practices domain (4 items) addressed awareness of protective mouthguards and their use during sports activities. All questions were closed-ended, except for parental and child age, which was categorized for analysis into several groups.

### 2.4. Data Analysis

All data were entered into Microsoft Excel and analyzed using IBM SPSS version 26.0. Descriptive statistics (frequencies and percentages) were used to summarize categorical variables. Knowledge scores were presented as means ± standard deviations or medians and interquartile ranges depending on normality (assessed by Kolmogorov–Smirnov test). Comparative analyses were performed between parents whose children had experienced dental trauma and those whose children had not. Chi-square tests were used to assess differences in categorical variables. The Mann–Whitney U test was used to examine differences in knowledge between parents whose child had experienced trauma and those whose child had not. To explore the relationship between sociodemographic factors and parental knowledge, a generalized linear regression model (GLM) was used. Independent variables included parental age, education, employment in healthcare, previous exposure to trauma education, and personal witnessing of trauma. The dependent variable was the total knowledge score. Results were reported using regression coefficients (beta) and 95% confidence intervals (CIs). Statistical significance was set at *p* < 0.05.

## 3. Results

Table 1 presents demographic and professional characteristics of the participating parents. A total of 333 parents participated in the study, including 65 males (19.5%) and 268 females (80.5%). A total of 145 participants (43.5%) reported that their children had experienced dental trauma, while 188 participants (56.5%) stated that their children had not experienced any dental trauma. The majority of participants were in the age range of 31–40 years, with 201 parents (60.3%) in this category. This group reported statistically significantly fewer instances of dental trauma in their children compared to the expected number, with only 76 parents (37.8%) indicating that their child had experienced trauma. In contrast, the younger group (≤30 years) reported more frequent instances of trauma, with 24 parents (70.6%) indicating that their child had experienced trauma. Participants from Zagreb reported significantly more frequent instances of dental trauma in their children compared to participants from Split. In Zagreb, 70 parents (51.9%) indicated that their child had experienced trauma, while in Split, 75 parents (37.9%) reported that their child had experienced trauma. No statistically significant differences were found regarding whether participants’ children had experienced dental trauma in relation to employment status and marriage status.

Table 2 displays the self-reported knowledge regarding dental injuries and their management at the site of the accident. Most of the respondents accurately identified that, in the case of a four-year-old child, dental trauma typically involves an injury of a deciduous tooth (99.1%), while in the case of a nine-year-old child, it typically involves a permanent tooth (97.6%). Only 55.3% of respondents correctly understood that an emergency procedure for an avulsed permanent tooth involves placing the tooth back into the dental alveolus. The rest either believed that the avulsed tooth should not be returned to the alveolus (12.6%) or were unsure of the correct answer (32.1%). 73.3% of respondents knew that it is important to visit a dentist immediately or within 30 min after an avulsion. Our participants had an average knowledge score of 7.68 out of a total possible score of 15. When knowledge scores were compared between parents whose children had experienced a traumatic dental injury and those without such experience, no statistically significant difference was observed (Mann–Whitney U Test, *p* = 0.185). Parents with prior experience were more likely to correctly recognize that trauma to primary teeth may affect the developing permanent teeth; however, this pattern was not consistent across other knowledge items.

Table 3 shows that most of the participants (51.1%) were partially informed about dental injuries during education. Most of them assess their personal knowledge as average (43.5%). Majority of them (68.5%) have witnessed traumatic tooth injury.

Table 4 shows that 145 (43.5%) parents reported that their child had experienced a dental trauma, most commonly occurring before the age of six (55.9%) and primarily caused by a fall (69%). The most common injuries were crown fracture (37.2%) and root fracture (28.9%), while luxation injuries (11.7%) and avulsion (10.3%) were less frequent. The most frequently affected teeth were the upper central incisor (76.6%) and lower central incisor (23.4%).

Table 5 shows that, although 54.3% of respondents were unaware of the purpose of dental shields, 82.3% considered them necessary for children during contact sports to prevent dental trauma. However, only 12.91% reported that their child wears a dental protector when participating in sports with a risk of tooth injury.

Table 6 presents the results of a linear regression analysis examining the association between parents’ knowledge of dental trauma and their personal and professional characteristics. Parents’ gender had a significant effect on knowledge, with mothers demonstrating higher levels of knowledge compared to fathers (*p* = 0.025). Furthermore, being informed about dental injuries during education was a strong predictor of knowledge. Parents who had been informed about dental trauma during their education showed significantly higher knowledge levels compared to those who had not been (*p* < 0.001), while partially informed parents also had higher knowledge scores (*p* = 0.002).

Additionally, parents who had witnessed a traumatic dental injury had significantly greater knowledge (*p* < 0.001). Other examined variables, including age, place of residence, educational level, marital status, employment, number of children, and socioeconomic status, did not show a statistically significant association with knowledge about dental trauma.

## 4. Discussion

The primary aim of this study was to evaluate the awareness of Croatian parents from Split and Zagreb regarding traumatic dental injuries and their management in children. The secondary aim was to assess the influence of previous TDI experience on parental knowledge. There was no statistically significant difference in knowledge between parents whose children had experienced dental trauma and those whose children had not. However, parents who had witnessed a dental trauma demonstrated better knowledge compared to those who had not, and mothers showed better knowledge than fathers. A study from Iran reported similar results, showing that mothers with previous experience of traumatic dental injuries demonstrated better self-reported performance [25]. This finding may be explained by the fact that mothers are generally more involved in their children’s daily care and health-related activities, including oral hygiene and dental visits. Furthermore, parents who have witnessed a dental trauma are likely to have better knowledge because direct exposure to such an event increases their awareness and motivation to learn how to manage similar situations in the future. Experiencing a real-life incident often triggers information seeking and reinforces learning through personal relevance, whereas parents who have not encountered dental trauma may lack the same sense of urgency or perceived need to acquire this knowledge. The findings indicated a moderate level of parental knowledge about the proper management of TDIs. A number of studies have assessed individuals’ knowledge levels about dental trauma and have shown that participants of various professions demonstrated a lack of knowledge about dental trauma [18,19,20,21,22,26].

A positive outcome of our study was that both groups—our participants and those examined in the Brazilian study—indicated that they would seek assistance from a dentist without delay. Although it was found that almost all parents would seek help from a dentist promptly in the event of an avulsion, unfortunately, the majority were not sufficiently informed about the appropriate way to preserve the tooth prior to reaching the dentist. A study conducted in Brazil showed that 60.92% of parents from public schools and 46.74% from private schools would preserve the tooth wrapped in paper or gauze, which is the least ideal medium for preserving an avulsed tooth. In our study, the most common response among participants was to store the avulsed tooth in saline solution, with 48.60% choosing this option. Meanwhile, 25.50% stated they would preserve the tooth in milk, which is considered a more suitable medium for maintaining the viability of an avulsed tooth. Milk is widely available and commonly found in most households, making it a practical and effective choice for emergency tooth preservation [18,27].

The findings of this study show that dental trauma is a very common issue among children, with 43.5% of parents reporting that their child had experienced a traumatic dental injury (TDI), most often before the age of six. This aligns with previous studies indicating a high frequency of TDIs in early childhood, a period characterized by underdeveloped coordination and increased risk of falls [4,5,28].

Falls were identified as the leading cause of dental injuries in our sample (69%), consistent with prior literature that highlights falls as the most frequent cause of dental trauma among children [3,5,17,28,29]. Studies have also shown that the home environment is a common setting for these injuries, rather than organized sports, although sports-related incidents become more prominent in older age groups [2,10]. Crown fractures (37.2%) and root fractures (28.9%) were the most reported injuries in our sample, while luxations (11.7%) and avulsions (10.3%) were less frequent. Similar patterns have been observed in previous studies, which confirm crown fractures as the most frequent type of injury [4,5,30].

The most commonly affected teeth were upper central incisors (76.6%), which are particularly vulnerable due to their anatomical position. This finding is widely supported in the literature, and it has significant clinical implications since injuries to these teeth can have long-term aesthetic and functional consequences, especially in younger children [4,5,16,17,30].

The findings indicate a significant gap between parental awareness and the actual use of dental mouthguards among children. While 82.3% of parents consider mouthguards necessary for contact sports, only 12.3% report that their child uses one. Similar studies have identified barriers such as lack of education, poor comfort, and limited encouragement from coaches and parents as key reasons for low compliance. Comprehensive educational initiatives and increased access to well-fitted mouthguards are essential to improve usage and prevent dental trauma in youth sports [21,31]. A concerning observation from both our study and the literature is the lack of knowledge among parents and other caregivers regarding the proper management of TDIs. Several recent studies continue to demonstrate low levels of awareness and knowledge, particularly when it comes to handling emergencies such as avulsion [7,8,9,14,15,18,20,32,33].

A cross-sectional study conducted in 2022 found that most mothers were unfamiliar with correct first aid for dental trauma, which may delay appropriate intervention and worsen prognosis [25]. Studies that have examined the impact of brief educational interventions on improving knowledge of emergency management of dental trauma have shown statistically significant improvements in knowledge following the intervention, with these gains also being retained over the long term. These findings suggest that the implementation of brief and straightforward educational programs could significantly improve knowledge within the general population. Emphasis should be placed on educating parents and schoolteachers, as they are most often the first to respond in cases of dental trauma involving children [26,34].

This study has several limitations that should be considered when interpreting the results. First, the sample was limited to parents attending two large dental polyclinics located in the two largest cities in Croatia, Split and Zagreb. This clinic-based sampling approach may be associated with selection bias and may limit the generalizability of the findings. Accordingly, the results should be interpreted as descriptive of parents attending dental clinics and should be extrapolated to the general Croatian parent population with caution. Parents who seek dental care might have higher health awareness and greater access to oral health information compared with those who do not regularly attend dental services. Additionally, it should be noted that there was a significantly higher number of female participants compared to male participants. There is a need for nationwide or multicenter studies to gather more representative data from a broader population across different regions and socioeconomic backgrounds in Croatia. This would provide a more comprehensive understanding of the gaps in knowledge and the barriers to effective management of dental injuries. Future studies should consider longitudinal designs to assess whether increased knowledge leads to better outcomes for children who experience traumatic dental injuries. Future research should address the limitations of the present study and further explore the topic of parental knowledge and management of TDIs through more extensive study designs. Longitudinal studies are recommended to assess changes in parental knowledge over time and to evaluate the long-term impact of educational interventions on the actual management and outcomes of TDIs.

Our findings emphasize the urgent need for better preventive measures and awareness campaigns. Recent reviews stress the importance of early intervention and public health strategies to mitigate the long-term consequences of TDIs [34]. In addition, training non-dental professionals, such as pediatricians, school staff, and coaches, has been identified as a promising strategy to improve the immediate response to injuries and reduce complications [19,20,34].

## 5. Conclusions

Considering the methodological limitations of the study, within the sample of parents attending dental clinics in Split and Zagreb, higher levels of knowledge and awareness of traumatic dental injuries were observed among female parents, those who had previously received education on dental trauma, and those with personal experience of such events. These findings suggest that educational exposure and firsthand experience may be associated with greater parental preparedness, although causal inferences cannot be made. Other sociodemographic characteristics were not significantly associated with knowledge levels, indicating that general demographic factors may be less informative predictors within this clinical context. Accordingly, targeted educational initiatives, particularly for parents without prior education or experience, may be beneficial, while broader generalization of these findings should be approached with caution.

## Figures and Tables

**Table 1:** Demographic and professional characteristics of the participating parents.

Characteristic of Parents		Total (*n* = 333)	Child Experienced Dental Trauma		*p*-Value
			Yes (*n* = 145)	No (*n* = 188)	
Gender	Male	65 (19.5)	46 (31.7)	19 (10.1)	<0.001
	Female	268 (80.5)	99 (68.3)	169 (89.9)	
Age	≤30	34 (10.2)	24 (16.6)	10 (5.3)	0.001
	31–40	201 (60.4)	76 (52.4)	125 (66.5)	
	>40 years	98 (29.4)	45 (31.0)	53 (28.2)	
Residence	Split	198 (59.5)	75 (51.7)	123 (65.4)	0.012
	Zagreb	135(40.5)	70 (48.3)	65 (34.6)	
Education	Primary Education	11 (3.3)	11 (7.6)	0 (0)	0.004
	Secondary Education	78 (23.4)	29 (20)	49 (26.1)	
	Bachelor’s or equivalent	83 (24.9)	35 (24.1)	48 (25.5)	
	Master’s or equivalent	135 (40.6)	59 (40.7)	76 (40.4)	
	Master of Science	18 (5.4)	9 (6.2)	9 (4.8)	
	Doctor of Philosophy	8 (2.4)	2 (1.4)	6 (3.2)	
Employment	Yes	290 (87.1)	127 (87.6)	163 (86.7)	0.811
	No	43 (12.9)	18 (12.4)	25 (13.3)	
Employed in medical/health profession	Yes	74 (22.2)	44 (30.3)	30 (16)	0.002
	No	259 (77.8)	101 (69.7)	158 (84)	
Socioeconomic status	Below average	13 (3.9)	11 (7.6)	2 (1.0)	<0.001
	Average	162 (48.7)	57 (39.3)	105 55.9)	
	Above average	158 (47.4)	77 (53.1)	81 (43.1)	
Marriage status	Unmarried	16 (4.8)	7 (4.8)	9 (4.8)	0.780
	Married	300 (90.1)	132 (91)	168 (89.4)	
	Divorced	17 (5.1)	6 (4.1)	11 (5.9)	
Number of children	1	103 (30.9)	45 (31)	58 (30.9)	<0.001
	2	162 (48.6)	57 (39.3)	105 (55.9)	
	3	59 (17.7)	42 (29.0)	17 (9).0	
	≥4	9 (2.7)	1 (0.7)	8 (4.3)	

Data are presented as the frequency (percentages). Data were tested by Chi-square or Fisher’s exact test (*p* < 0.05).

**Table 2:** Distribution of parents’ responses regarding their knowledge of dental injuries and management at the place of the accident.

Question	Answer	Total (*n* = 333)	Child Experienced Dental Trauma		*p*-Value
			Yes (*n* = 145)	No (*n* = 188)	
Front teeth (incisor) injuries in a 4-year-old usually include	Permanent teeth	1 (0.3)	0 (0)	1 (0.5)	0.311
	*Deciduous teeth*	330 (99.1)	145 (100)	185 (98.4)	
	I do not know	2 (0.6)	0 (0)	2 (1.1)	
Front teeth (incisor) injuries in a 9-year-old usually include	*Permanent teeth*	325 (97.6)	142 (97.9)	183 (97.3)	0.439
	Deciduous teeth	6 (1.8)	3 (2.1)	3 (1.6)	
	I do not know	2 (0.6)	0 (0)	2 (1.1)	
Type of care sought by parents if their child experiences a bleeding dental trauma	Ambulance	68 (20.4)	25 (17.2)	43 (22.8)	0.412
	General practitioner or pediatrician	3 (0.9)	1 (0.7)	2 (1.1)	
	*Dentist*	262 (78.7)	119 (82.1)	143 (76.1)	
Emergency treatment for a fractured tooth at the site of the accident	Seek the help of a dentist, and do not look for the broken part of the tooth because it is useless	80 (24)	37 (25.5)	43 (22.9)	0.717
	Find the broken part of the tooth, wrap it in a handkerchief, and seek the help of a dentist	94 (28.2)	42 (29)	52 (27.6)	
	*Find the broken part of the tooth, put it in a liquid medium, and seek the help of a dentist*	122 (36.6)	53 (36.5)	69 (36.7)	
	I do not know	37 (11.1)	13 (9)	24 (12.8)	
Best emergency treatment for tooth displacement at the site of the accident	*Try to return the tooth to its original position*	17 (5.1)	9 (6.2)	8 (4.2)	0.876
	*Ask the injured person to grit his teeth if it is possible*	20 (6)	9 (6.2)	11 (5.9)	
	Do not touch the tooth, leave it in this position until you come to the dentist	234 (70.3)	100 (69)	134 (71.3)	
	I do not know	62 (18.6)	27 (18.6)	35 (18.6)	
Emergency procedure for an avulsed deciduous tooth involves replantation into the alveolus	Yes	70 (21)	36 (24.8)	34 (18.1)	0.313
	*No*	159 (47.7)	67 (46.2)	92 (48.9)	
	I do not know	104 (31.2)	42 (29)	62 (33)	
Emergency procedure for an avulsed permanent tooth involves replantation into the alveolus	*Yes*	184 (55.3)	75 (51.7)	109 (58)	0.469
	No	42 (12.6)	21 (14.5)	21 (11.2)	
	I do not know	107 (32.1)	49 (33.8)	58 (30.8)	
Trauma to deciduous teeth can affect the permanent tooth germ	No	51 (15.3)	15 (10.3)	36 (19.1)	0.018
	*Yes*	182 (54.7)	91 (62.8)	91 (48.4)	
	I do not know	100 (30)	39 (29.9)	61 (32.5)	
Procedure for cleaning a contaminated tooth before replantation on site of accident	Disinfect with alcohol	61 (18.3)	28 (19.3)	33 (17.6)	0.454
	Clean with a toothbrush and disinfectant	55 (16.5)	23 (15.9)	32 (17)	
	*Rinse with cold tap water*	58 (17.4)	23 (15.9)	35 (18.6)	
	Without cleaning return to alveolus	2 (0.6)	1 (0.7)	1 (0.5)	
	Clean with a brush and toothpaste	18 (5.4)	12 (8.2)	6 (3.2)	
	I do not know	139 (41.7)	58 (40)	81 (43.1)	
Storage medium for an avulsed tooth if immediate replantation is not possible (multiple responses allowed)	*Special tooth storage medium*	35 (10.5)	13 (9)	22 (11.7)	0.955
	*Milk*	85 (25.5)	41 (28.3)	44 (23.4)	
	*Saliva-mouth of child*	67 (20.1)	31 (21.4)	36 (19.1)	
	Handkerchief	26 (7.8)	11 (7.6)	15 (8)	
	Ice	19 (5.7)	9 (6.2)	10 (5.3)	
	Alcohol	9 (2.7)	3 (2.1)	6 (3.2)	
	Saline solution	162 (48.6)	70 (48.3)	92 (48.9)	
	I do not know	71 (21.3)	30 (20.7)	41 (21.8)	
Handling technique for an avulsed tooth	By the root	83 (24.9)	35 (24.1)	48 (25.6)	0.873
	*By the crown*	93 (27.9)	39 (26.9)	54 (28.7)	
	Never mind	6 (1.8)	2 (1.4)	4 (2.1)	
	I do not know	151 (45.3)	69 (47.6)	82 (43.6)	
Ideal time to seek professional care for a tooth avulsion	*Immediately*, *within the first 30 min from the injuries*	244 (73.3)	107 (73.8)	137 (72.9)	0.302
	Within a few hours	79 (23.7)	36 (24.8)	43 (22.9)	
	Next day	0 (0)	0 (0)	0 (0)	
	I do not know	10 (3)	2 (1.4)	8 (4.2)	

Data are presented as the frequency (percentages). Differences were analyzed using Chi-square or Fisher’s exact test (*p* < 0.05). Correct answers are in italics.

**Table 3:** Parents’ awareness and experience of dental trauma.

Characteristic		Total (*n* = 333)	Child Experienced Dental Trauma		*p*-Value
			Yes (*n* = 145)	No (*n* = 188)	
Informed about dental injuries during education (academic or professional).	Yes	70 (21)	52 (35.9)	18 (9.6)	<0.001
	Partiallyinformed	170 (51.1)	61 (42.2)	109 (58)	
	No	93 (27.9)	32 (22.1)	61 (32.4)	
Assess personal knowledge of preventive and therapeutic procedures for traumatic dental injuries:	Very bad	32 (9.6)	10 (6.9)	22(11.7)	0.082
	Bad	99 (29.7)	46 (31.7)	53 (28.2)	
	On average	145 (43.5)	62 (42.8)	83 (44.1)	
	Good	40 (12)	23 (15.9)	17 (9)	
	Very good	17 (5.1)	4 (2.8)	13 (6.9)	
Witnessed a traumatic tooth injury.	No	105 (31.5)	10 (6.9)	95 (50.5)	<0.001
	Yes	228 (68.5)	135 (93.1)	93 (49.5)	

Data are presented as the frequency (percentages). Data were tested by Chi-square or Fisher’s exact test (*p* < 0.05).

**Table 4:** Parents’ personal experience regarding children’s dental trauma.

Question	Answer	Total (*n* = 145)
Gender of child	Male	75 (51.72)
	Female	70 (48.28)
Age (years) of child at the time of dental trauma	≤6	81 (55.9)
	7–8	17 (11.7)
	9–12	21 (14.5)
	>12	26 (17.9)
Type of tooth injury	Crown fracture	54 (37.2)
	Root fracture	42 (28.9)
	Dislodged tooth, not completely avulsed	17 (11.7)
	Avulsion (completely knocked-out tooth)	15 (10.3)
	Other	17 (11.7)
Type of injured tooth	Deciduous tooth	88 (60.7)
	Permanent tooth	57 (39.3)
Position of injured tooth	Upper central incisor	111 (76.6)
	Lower central incisor	34 (23.4)
	Upper lateral incisor	0 (0)
	Lower lateral incisor	0 (0)
Sought dental help for the injury	No	19 (13.1)
	Yes	126 (86.9)
Cause of dental injury	Fall	100 (69)
	Sports	10 (6.9)
	Game	19 (13.1)
	Traffic	0 (0)
	Violence	0 (0)
	Illness	16 (11)
	Other	0 (0)
Place of incident	Playground	16 (11)
	Kindergarten	15 (10.4)
	Classroom	12 (8.3)
	House	37 (25.5)
	Street	37 (25.5)
	Other	28 (19.3)
Time of day when the accident occurred	Morning	41 (28.3)
	Afternoon	58 (40.0)
	Evening	34 (23.4)
	I do not know	12 (8.3)
Season when the accident occurred	Spring	20 (13.8)
	Summer	34 (23.4)
	Autumn	24 (16.6)
	Winter	63 (43.4)
	I do not know	4 (2.8)
Source of information about dental trauma (multiple responses allowed)	Dentist	207 (62.2)
	Pediatrician or school doctor	47 (14.1)
	Internet portals and forums	114 (34.2)
	Family members, friends, colleagues	117 (35.1)
	I am not informed	58 (17.4)

Data are presented as the frequency (percentages).

**Table 5:** Parents self-reported knowledge regarding dental mouthguard.

Question	Answer	Total (*n* = 333)	Child Experienced Dental Trauma		*p*-Value
			Yes (*n* = 145)	No (*n* = 188)	
Familiarity with the use of dental mouthguards	Yes	155 (46.5)	73 (50.3)	82 (43.6)	0.222
	No	178 (53.4)	72 (49.7)	106 (56.4)	
Opinion on children wearing dental mouthguards during sports to prevent dental trauma	No	59 (17.7)	25 (17.2)	34 (18.1)	0.842
	Yes	274 (82.3)	120 (82.8)	154 (81.9)	
My child wears a dental mouthguard during sports at risk of tooth injury	Yes	41 (12.9)	20 (13.8)	21 (11.2)	0.470
	No	292 (87.1)	125 (86.2)	167 (88.8)	
Type of dental guard used by your child	Commercial (available in sporting goods stores)	21 (6.3)	10 (47.6)	11 (50)	0.283
	Semi-individual (“cook and bite”)	17 (5.1)	9 (42.9)	8 (36.4)	
	Individual (made from jaw print)	5 (1.5)	2 (9.5)	3 (13.6)	

Data are presented as the frequency (percentages). Data were tested by Chi-square or Fisher’s exact test (*p* < 0.05).

**Table 6:** Linear regression analysis of factors associated with parents’ knowledge and awareness regarding traumatic dental injuries.

Characteristic		β (95% CI)	*p*-Value
Parents gender	Male	Reference	
	Female	0.959 (0.122–1.796)	0.025
Parents age	<30	Reference	
	31–40	0.577 (−0.535–1.688)	0.309
	>40	0.179 (−1.043–1.402)	0.774
Place of residence	Split	Reference	
	Zagreb	−0.442 (−1.025–0.141)	0.137
Academic degree	Primary Education	Reference	
	Secondary Education	0.229 (−4.944–5.402)	0.931
	Bachelor’s or equivalent	−0.055 (−5.239–5.129)	0.983
	Master’s or equivalent	1.153 (−4.056–6.363)	0.664
	Master of Science	0.879 (−4.466–6.224)	0.747
	Doctor of Philosophy	4.048 (−1.367–9.462)	0.143
Employment	Yes	0.587 (−0.347–1.521)	0.218
	No	Reference	
Employed in medical/health profession	Yes	0.398 (−0.567–1.364)	0.419
	No	Reference	
Socioeconomic status	Below average	Reference	
	Average	−1.757 (−6.391–2.877)	0.457
	Above average	−0.219 (−4.958–4.519)	0.928
Marriage status	Unmarried	−1.030 (−2.259–0.199)	0.101
	Married	Reference	
	Divorced	0.124 (−1.196–1.445)	0.853
Number of children	1	Reference	
	2	0.095 (−0.611–0.801)	0.792
	3	−0.205 (−1.097–0.688)	0.653
	4	−1.302 (−3.022–0.417)	0.138
Informed about dental injuries during education	Yes	3.544 (2.118–4.970)	<0.001
	Partially informed	1.164 (0.433–1.895)	0.002
	No	Reference	
Self-assessment of knowledge on preventive and therapeutic procedures for dental trauma	Very bad	Reference	
	Bad	−0.146 (−1.213–0.921)	0.788
	Average	0.212 (−0.927–1.351)	0.715
	Good	0.025 (−1.530–1.580)	0.975
	Very good	1.340 (−0.413–3.094)	0.134
Witnessed a traumatic tooth injury	No	Reference	
	Yes	1.381 (0.747–2.016)	<0.001
Child experienced dental trauma	No	Reference	
	Yes	−0.538 (−1.185–0.109)	0.103

Data are presented as numbers. The reference knowledge category is “low”. β, correlation coefficient; 95% CI, 95% confidence interval. *p* ≤ 0.05.

## Data Availability

The data are available from the corresponding author upon reasonable request.
